# Chronic airway disease as a major risk factor for fractures in osteopenic women: Nationwide cohort study

**DOI:** 10.3389/fendo.2023.1085252

**Published:** 2023-03-21

**Authors:** Sung Hye Kong, Ae Jeong Jo, Chan Mi Park, Kyun Ik Park, Ji Eun Yun, Jung Hee Kim

**Affiliations:** ^1^ Department of Internal Medicine, Seoul National University Bundang Hospital, Seongnam, Republic of Korea; ^2^ Department of Internal Medicine, Seoul National University College of Medicine, Seoul, Republic of Korea; ^3^ Department of Information Statistics, Andong National University, Kyongbuk, Republic of Korea; ^4^ Department of Health Technology Assessment, National Evidence-Based Healthcare Collaborating Agency (NECA), Seoul, Republic of Korea; ^5^ Department of Internal Medicine, Seoul National University Hospital, Seoul, Republic of Korea

**Keywords:** osteopenia, nationwide, cohort, fractures, cardiovascular disease, cerebrovascular disease, asthma

## Abstract

**Introduction:**

The study aimed to demonstrate the risk factors for fractures and to develop prediction models for major osteoporotic and hip fractures in osteopenic patients using the nationwide cohort study in South Korea.

**Methods:**

The study was a retrospective nationwide study using the national screening program for transitional ages from the National Health Insurance Services database in Korea from 2008 to 2019. Primary outcomes were incident fracture events of major osteoporotic and hip fractures. Major osteoporotic and hip fracture events were defined as diagnostic and procedural codes. Patients were followed until the fragility fractures, death, or 2019, whichever came first.

**Results:**

All participants were 66-year-old females, with a mean body mass index was 25.0 ± 3.1 kg/m^2^. During a median follow-up of 10.5 years, 26.9% and 6.7% of participants experienced major osteoporotic and hip fractures. In multivariate analysis, a history of fracture, chronic airway disease, falls, diabetes mellitus and cerebrovascular diseases were significant risk factors for major osteoporotic (hazard ratio [HR] 2.35 for a history of fracture; 1.17 for chronic airway disease; 1.10 for falls; 1.12 for diabetes mellitus; 1.11 for cerebrovascular disease) and hip fractures (HR 1.75 for a history of fracture; 1.54 for diabetes mellitus; 1.27 for cerebrovascular disease; 1.17 for fall; 1.15 for chronic airway disease). The performances of the prediction models were area under the receiver operating curve of 0.73 and 0.75 for major osteoporotic and hip fractures.

**Conclusion:**

The study presented prediction models of major osteoporotic and hip fractures for osteopenia patients using simple clinical features.

## Introduction

The treatment is cost-effective for patients with osteoporosis, defined according to the World Health Organization criterion, with low bone mineral density (BMD T-score of -2.5 or less), or a history of a fragility fracture, as previously reported (1). However, there is little consensus on when to start treatment in patients with osteopenia. Osteopenia, a subclinical condition of low bone mass with a T-score between -1.0 and -2.5, is significantly more common than osteoporosis in South Korea and the US and accounts for more than half of patients with fragility fractures (2–4). Considering these factors, some patients with osteopenia may warrant treatment, and it is also essential to determine the high-risk patients among them.

Thus, there is a practical need for an individualized assessment of fracture risk in patients with osteopenia. Although the fracture risk assessment tool (FRAX) and Garvan fracture risk models help to predict fracture risk (5), they tend to underestimate the risk in low-risk patients in some studies (5–8). Furthermore, while various diseases such as secondary osteoporosis are included as risk factors in FRAX, chronic diseases such as cardiovascular and cerebrovascular diseases, type 2 diabetes mellitus, and a history of falls, which are critical risk factors for fractures, were not included. In addition, bone density alone offers limited predictive power in osteopenic patients (9). Hence, to identify patients with osteopenia with a high risk of fracture before the bone density worsens, it is necessary to assess known risk factors such as chronic diseases (4, 10). Therefore, it is essential to design a new fracture prediction model and associated risk factors according to fracture types for patients with osteopenia. Herein, the study aimed to identify additional risk factors for fractures and to develop prediction models for major osteoporotic and hip fractures in patients with osteopenia using the nationwide cohort study in South Korea.

## Methods

### Data source

This retrospective nationwide study was conducted using the information retrieved from National Health Insurance Services (NHIS) database of South Korea from 2008 to 2019. This insurance system by the Korean government covers approximately 97.2% of Korean residents and contains data on healthcare services reimbursed including demographics, diagnoses, prescriptions, diagnostic or surgical procedures, and medical costs. The national screening program for transitional ages (NSPTA) launched in 2007, by the Korean government, conducts BMD testing for 66-year-old women (11). BMD was primarily measured using dual-energy X-ray absorptiometry (DXA) at the spine or at the femoral neck, if it was not possible to measure at the spine due to vertebral fracture or surgery (12). Every individual was anonymized using a personal identification number, which enabled the longitudinal follow-up. The Institutional Review Board of the National Evidence-based healthcare Collaborating Agency (NECA) (No.NECAIRB20-004) approved the study protocol, and the requirement for informed consent was waived-off as the patient information was anonymized. The study was funded by the National Research Foundation of Korea (NRF) and NECA.

### Study population

**Figure 1:**
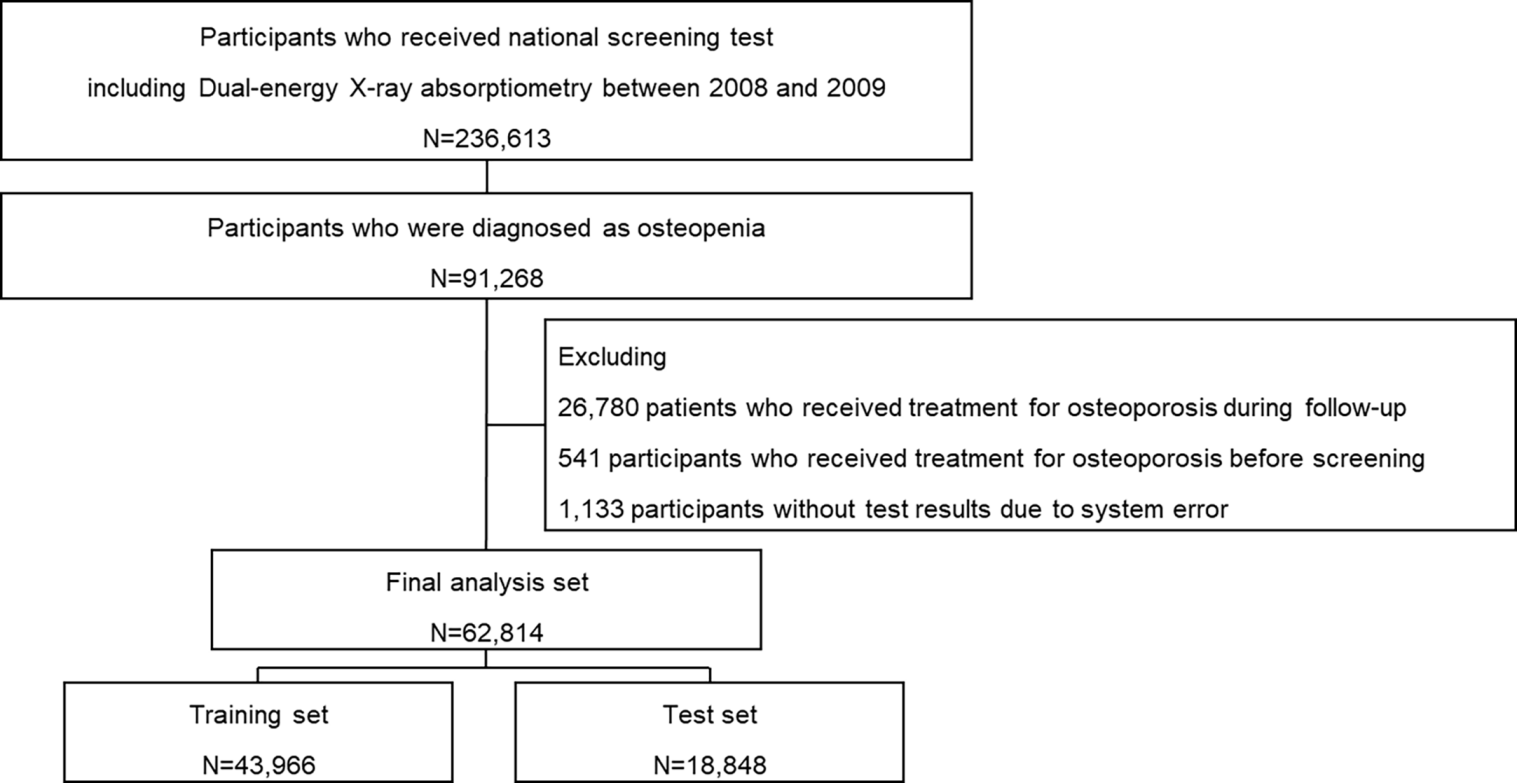
Selection of study participants.

A total of 236,582 individuals received NSPTA health examinations at the age of 66 between January 1, 2008, and December 31, 2009. They were followed up until December 31, 2019, to ensure a maximum of 10 years of follow-up. Among them, 91,268 individuals diagnosed with osteopenia were initially selected. From the selected individuals, 26,780 who received treatment for osteoporosis during follow-up (bisphosphonate, denosumab, teriparatide, or romosozumab), 541 who received treatment for osteoporosis before the screening, and 1,133 without test results due to system error were excluded from the study. A total of 62,814 individuals were included in the final analysis set (**Figure 1**). The study participants were randomly split into 7:3 training and test sets. The cohort entry date was defined as the date of BMD screening. A year prior to the entry date of the cohort was used to determine study eligibility and baseline clinical characteristics.

### Operational definition of primary outcomes and comorbidities

The major osteoporotic and hip fractures events, defined by the diagnostic codes of the 10th version of the International Classification of Diseases (ICD-10), occurred during the follow-up period were considered the primary outcomes. The major osteoporotic fracture events were defined as hospital visits of ≥2 times due to the diagnostic codes (S22.0, S22.1, S32.0, M48.4, M48.5, S42.2, S42.3, S52.5, and S52.6) from admission or outpatient department after the index date or hip fracture. Hip fracture events were defined as more than one hospital visits due to the diagnostic codes (S72.0, S72.1) from admission or outpatient department with more than one treatment codes (N0601, N0611, N0305, N0981, N0641, N0652, N0654, N0711, N0715) after the index date. The follow up period was from the date of cohort entry to the occurrence of fragility fractures, death, or end of the study period (December 31, 2019), whichever came first.

Body mass index (BMI) was measured at their entry date. The history of falls, social history (smoking and drinking), and physical activity information was collected using the standardized self-administered questionnaires. Ever smoker was defined as the participants who were ex-smokers and current smokers and drinker as participants who drank alcohol more than once per week.

Physical activity at baseline examination was analyzed using the International Physical Activity Questionnaire (IPAQ), which assessed three domains: the mode, frequency, and intensity of the activity. The survey questionnaire included the number of days of physical activity in a week during the past six months. Physical activity of at least 30 min/day was categorized based on the frequency of the activity (times/week) as 0 times/week: Q1, 1-2 times/week: Q2, 3-5 times/week: Q3, 6-7 times/week: Q4. Additionally, it was classified based on the intensity of the activity (walking, moderate, or vigorous). Moderate physical activity was defined as a slight increase in breathing or heart rate or fairly-hard perceived exertion, such as carrying light loads, slow cycling, and fast walking. Vigorous physical activity was defined as a substantial increase in breathing or heart rate or in moderately-hard perceived exertion, such as carrying heavy loads, fast cycling, running, mountain climbing, playing soccer, or any other activity. Moderate-to-vigorous physical activity was defined in this study as moderate or vigorous physical activity more than once/week during the past 6 months.

History of fractures, diabetes mellitus, cardiovascular diseases, cerebrovascular diseases, chronic renal failure, and chronic airway diseases (asthma/chronic obstructive pulmonary diseases (COPD)) were determined by diagnostic codes. To ensure an accurate diagnosis, diabetes mellitus (E10-E14), cardiovascular diseases (I20-I22), cerebrovascular diseases (I63, I64, I693, I694, G45, I60-62, I690-692), chronic renal failure (N183, N184, N185, N258, Z491, Z492, Z940), and chronic airway disease including asthma/COPD (J45) were regarded as present if a participant was treated ≥2 times. Steroid users were defined as participants who had chronic exposure to glucocorticoids (≥5 mg of prednisolone-equivalent steroid/day for ≥3 months). Secondary causes for osteopenia were defined as type 1 diabetes, osteogenesis imperfecta in adults, hyperthyroidism, hypogonadism, premature menopause (<45 years), chronic malnutrition, malabsorption, and chronic liver disease (5).

Additionally, laboratory findings such as hemoglobin and liver enzymes levels were considered risk factors for osteoporotic fractures. Quality control procedures of laboratory data complied with the Korean Association of Laboratory Quality Control guidelines. Hemoglobin levels were categorized as desirable (≥15.5 g/dL), borderline-low (12–15.49 g/dL), and low (<12 g/dL). Gamma glutamate transferase (GGT) values were classified as normal (<35 U/L), and abnormal (≥35 U/L). Total cholesterol values were classified as normal (≤200 mg/dL), and abnormal (>200 mg/dL).

### Statistical analyses

The cohort data was randomly stratified into two groups: 70% random sampling for the model development and 30% for validation. Continuous data were presented as mean ± standard deviation, and categorical data were reported as actual numbers (%). Participant characteristics in both groups were compared using Student’s *t*-test for continuous variables and the χ^2^ test for categorical variables. The risk factors considered in the initial model were body mass index, history of falls, smoking status, alcohol drinking, physical activity, comorbidity, history of fractures, concomitant drugs used, and laboratory data, including hemoglobin, cholesterol, and GGT levels. Univariate analyses were used to regress the sub-distribution hazard of osteoporotic fracture incidence on all candidate variables.

Cox proportional hazard regression models were used to estimate β coefficient, hazard ratios (HRs), and 95% confidence intervals (CIs) of major osteoporotic and hip fractures, considering death as a competing risk using the Fine and Gray model (13). Variable selection was performed using a multivariate model to build a risk prediction model. The Cox models assigned risk scores based on HR for each risk factor. Considering significant covariates from univariate analysis and variables with clinical importance, three models confirmed to fit through the Hosmer-Lemeshow test, a statistical test for the fit of the model (**Supplementary Tables 1**, **2**). Among them, the model with the highest discriminatory power, assessed by the area under the receiver operating curve (AUROC) was selected. The predictive models were estimated by applying the risk function calculated through the cumulative incidence curve. Survival time was calculated from cohort entry until the occurrence of primary outcomes or until December 31, 2019, whichever came first. The performance of the developed model was tested through the validation dataset. All analyses were conducted using SAS, version 9.4 (SAS Institute Inc., Cary, NC) and R, version 3.4.3 (R Foundation for Statistical Computing, Vienna, Austria).

## Results

### Clinical characteristics

The clinical characteristics of 62,814 participants (training set: 43,966 and test set: 18,848) are presented in **Table 1**. All the participants were 66-year-old females with a mean body mass index of 25.0 ± 3.1 kg/m^2^. Among them, 6,613 (10.5%) experienced falls and 1,332 (2.1%) had a history of osteoporotic fractures and 27,637 (24.4%) did moderate-to-vigorous physical activity. The frequency of the participants with co-morbidities at the baseline were: diabetes mellitus (24.4%), cardiovascular diseases (9.1%), cerebrovascular diseases (5.7%), cancer (2.3%), chronic renal failure (0.3%), and chronic airway disease (8.9%). Among the participants, 786 (1.2%) were long-term steroids users. The baseline characteristics were similar between training and test sets. During a median follow-up of 10.5 years (range 1.0–12.0), major osteoporotic and hip fracture events occurred in 17,265 (26.9%) and 4,284 (6.7%) cases, respectively (**Supplementary Figure 1**).

**Table 1:** Clinical characteristics of patients with osteopenia.

	Total		Training set		Test set		*p*
	(n=62,814)		(n=43,996)		(n=18,848)		
**Body mass index**	25.0	± 3.1	25.0	± 3.1	25.0	± 3.1	0.406
**Ever smoker**	1,271	(2.0)	893	(2.0)	378	(2.0)	0.834
**Current drinker**	6,853	(10.9)	4,797	(10.9)	2,056	(10.9)	0.993
**Low physical activity**	35,177	(56.0)	24,651	(56.0)	10,556	(56.0)	0.989
**History of fall**	6,613	(10.5)	4,612	(10.5)	2,001	(10.6)	0.635
**Diabetes mellitus**	15,329	(24.4)	10,668	(24.3)	4,661	(24.7)	0.213
**Cardiovascular disease**	5,736	(9.1)	4,045	(9.2)	1,691	(9.0)	0.362
**Cerebrovascular disease**	3,608	(5.7)	2,527	(5.7)	1,081	(5.7)	0.951
**Chronic kidney disease**	212	(0.3)	142	(0.3)	70	(0.4)	0.337
**Epilepsy**	439	(0.7)	309	(0.7)	130	(0.7)	0.856
**Dementia**	659	(1.0)	458	(1.0)	201	(1.1)	0.780
**Chronic airway diseases**	5,631	(9.0)	3,951	(9.0)	1,680	(8.9)	0.768
**Idiopathic hypercalciuria**	213	(0.3)	137	(0.3)	76	(0.4)	0.070
**Secondary causes for osteopenia**	2,164	(3.4)	1,499	(3.4)	665	(3.5)	0.454
**History of fracture**	1,332	(2.1)	935	(2.1)	397	(2.1)	0.871
**Use of steroid**	786	(1.3)	555	(1.3)	231	(1.2)	0.704
**Hemoglobin, mg**	12.9	± 1.1	12.9	± 1.1	12.9	± 1.1	0.273
**Total cholesterol, mg/dL**	206.1	± 42.9	206.1	± 40.3	206.2	± 48.4	0.785
**γ-GGT, mg/dL**	26.3	± 30.3	26.3	± 30.9	26.1	± 28.6	0.465

GGT, Glutamyl transpeptidase. Every patient was 66-year-old woman due to the date characteristics. Low physical activity was defined as patients who do not have moderate or vigorous physical activity during the past 6 months. Chronic airway diseases include asthma or chronic obstructive pulmonary disease. Use of steroids was defined as patients who have been exposed to oral glucocorticoids for more than 3 months at a dose of prednisolone of 5mg daily or more during the past year. Continuous variables are expressed as mean ± standard deviation and categorical variables as numbers (percentages). Comparisons between groups were analyzed by Student t-test for continuous variables and χ^2^ test for categorical variables.

### Factors associated with major osteoporotic and hip fractures

The participants with a history of falls had 1.23 and 1.38-times increased risk and with a history of a previous fracture had 2.33 and 2.25-times higher risk, for major osteoporotic and hip fractures (**Table 2**). A high level of GGT increased risk of both major osteoporotic and hip fractures. Diabetes mellitus, cardiovascular and cerebrovascular diseases, chronic airway disease, secondary causes for osteopenia, and use of steroids, were other common risk factors.

**Table 2:** Risk factors for major osteoporotic and hip fractures in osteopenia patients.

Variables	Major osteoporotic fracture				Hip fracture			
	HR	95% CI		*p*	HR	95% CI		*p*
		Lower	Higher			Lower	Higher	
**BMI < 18.5 kg/m^2^**	Ref				Ref			
**≥ 18.5, <25 kg/m^2^**	0.89	0.75	1.06	.206	1.13	0.78	1.64	.499
**≥ 25 kg/m^2^**	0.91	0.76	1.08	.299	1.27	0.88	1.84	.197
**Ever smoker**	1.12	0.99	1.25	.051	1.36	1.12	1.66	.001
**Current drinker**	1.08	1.03	1.14	.001	1.15	1.04	1.27	.003
**Moderate-to-vigorous** **physical activity**	Ref				Ref			
**Low physical activity**	1.04	1.01	1.08	.006	0.94	0.88	1.00	.091
**History of fall**	1.22	1.16	1.29	<.001	1.27	1.16	1.40	<.001
**Diabetes mellitus**	1.15	1.11	1.19	<.001	1.55	1.45	1.66	<.001
**Cardiovascular disease**	1.08	1.03	1.15	.002	1.25	1.13	1.39	<.001
**Cerebrovascular disease**	1.13	1.06	1.21	<.001	1.36	1.20	1.53	<.001
**Chronic kidney disease**	0.99	0.73	1.34	.969	1.77	1.13	2.75	.011
**Epilepsy**	1.19	0.99	1.43	.063	1.39	0.99	1.94	.051
**Dementia**	1.20	1.03	1.40	.018	1.28	0.96	1.70	.090
**Chronic airway diseases**	1.20	1.14	1.27	<.001	1.25	1.13	1.39	<.001
**Idiopathic hypercalciuria**	1.17	0.87	1.55	.281	1.52	0.94	2.44	.082
**Secondary causes for osteopenia**	1.18	1.08	1.29	<.001	1.23	1.05	1.45	.010
**History of fracture**	2.45	2.24	2.69	<.001	2.09	1.77	2.47	<.001
**Use of steroid**	1.61	1.41	1.84	<.001	1.54	1.21	1.96	<.001
**Hemoglobin < 12 g/dL**	Ref				Ref			
**≥ 12, < 15.5 g/dL**	1.02	0.83	1.24	.837	0.93	0.64	1.36	.722
**≥ 15.5 g/dL**	1.08	0.88	1.33	.419	1.00	0.68	1.47	.974
**Total cholesterol > 200 mg/dL**	Ref				Ref			
**≤ 200 mg/dL**	0.94	0.91	0.97	<.001	0.95	0.89	1.01	.151
**γGTP ≤ 35 mg/dL**	Ref				Ref			
**> 35 mg/dL**	1.05	1.01	1.10	.013	1.16	1.07	1.27	<.001

HR, hazard ratio; CI, confidence interval; GTP, Glutamyl transpeptidase. Low physical activity was defined as patients who do not have moderate or vigorous physical activity during the past 6 months. Chronic airway diseases include asthma or chronic obstructive pulmonary disease. Use of steroids was defined as patients who have been exposed to oral glucocorticoids for more than 6 months at a dose of prednisolone of 5mg daily or more during the past year. Univariate Cox regression analyses were done.

The participants with a history of drinking had increased risk of major osteoporotic fractures. In addition, participants who had low physical activity in their daily routine had an increased risk of major osteoporotic fracture than those with low physical activity, but were not associated with hip fractures. Participants with chronic kidney disease also had an increased risk of hip fractures. However, history of drinking, moderate-to-vigorous physical activity, and history of chronic kidney diseases were found to be insignificant in multivariate analysis.

### Prediction models for major osteoporotic and hip fractures in osteopenia

The variables that showed statistical significance in univariate analysis were introduced into multivariate analysis to develop the prediction models (**Supplemental Tables 1**, **2**). In the multivariate analysis, a history of fracture (HR=2.35, 95% CI=2.14–2.58), chronic airway disease (HR=1.17, 95% CI=1.12–1.24), fall (HR=1.10, 95% CI=1.04–1.16), diabetes mellitus (HR=1.12, 95% CI=1.08 – 1.16), and cerebrovascular disease (HR=1.11, 95% CI 1.03–1.19) showed significance. After the selection process to derive the model with the best performance, a history of fracture, chronic airway disease, fall, diabetes mellitus, and cerebrovascular diseases remained major contributing factors (**Table 3**). The model showed AUROCs of 0.732 and 0.726 in training and test sets, respectively.

**Table 3:** Prediction model for major osteoporotic fracture in osteopenia patients.

Variables	Beta-coefficient	HR	95% CI	*p*
**History of fracture**	0.848	2.34	2.12 – 2.57	<.001
**Chronic airway diseases**	0.178	1.19	1.13 – 1.26	<.001
**History of fall**	0.130	1.14	1.08 – 1.20	<.001
**Diabetes mellitus**	0.127	1.14	1.09 – 1.18	<.001
**Cerebrovascular disease**	0.101	1.11	1.03 – 1.18	0.004
**P for Wald’s test**	<0.001			
**AUC of the training set**	0.732			
**AUC of the test set**	0.726			

HR, hazard ratio; CI, confidence interval; AUC, area under the curve. Cox regression analyses were done. Multivariate analyses were adjusted for history of fracture, asthma/COPD, falls, diabetes mellitus, and cerebrovascular disease.


$$h(t)=h_{0}(t)e\;(0.130\;\times \;history\;of\;fall\;+\;0.125\;\times \;diabetes\;mellitus\;+\;0.099\;\times \;cerebrovascular\;disease\;+\;0.180\;\times \;chronic\;airway\;disease\;+\;0.850\;\times \;history\;of\;fracture),$$

where *h*_0_(10)=0.279.

The multivariate analysis for hip fracture showed significance for a history of fracture (HR=1.75, 95% CI=1.46–2.10), diabetes mellitus (HR=1.54, 95% CI=1.43–1.65), smoking (HR=1.25, 95% CI=1.01–1.53), cerebrovascular disease (HR=1.27, 95% CI=1.12–1.43), fall (HR=1.17, 95% CI=1.06–1.30), and chronic airway disease (HR=1.15, 95% CI=1.04–1.28). After the selection process to derive the model with the best performance, a history of fracture, diabetes mellitus, cerebrovascular disease, chronic airway disease, and falls remained major contributing factors for hip fractures (**Table 4**). The model showed AUROCs of 0.743 and 0.745 in training and test sets, respectively.

**Table 4:** Prediction model for hip fracture in osteopenia patients.

Variables	Beta-coefficient	HR	95% CI	*p*
**History of fracture**	0.650	1.92	1.62 – 2.27	<0.001
**Diabetes mellitus**	0.416	1.52	1.41 – 1.63	<0.001
**Cerebrovascular disease**	0.230	1.26	1.11 – 1.42	<0.001
**Chronic airway diseases**	0.197	1.22	1.09 – 1.35	<0.001
**History of fall**	0.171	1.19	1.08 – 1.31	<0.001
**P for Wald’s test**	<0.001			
**AUC of the training set**	0.743			
**AUC of the test set**	0.745			

HR, hazard ratio; CI, confidence interval; AUC, area under the curve. Cox regression analyses were done. Multivariate analyses were adjusted for history of fracture, diabetes mellitus, cerebrovascular disease, asthma/COPD, and history of falls.


$$h\left(t\right)=\;h_{0}\left(t\right)e\;(0.175\times history\;of\;fall+0.407\times diabetes\;mellitus+0.228\times cerebrovascular\;disease+0.193\times chronic\;airway\;disease+0.656\times history\;of\;fracture),$$

where *h*_0_(10)=0.064.

## Discussion

The data from the Korean nationwide cohort of 66-year-old women with osteopenia, showed that 26.9% and 6.7% of the participants experienced major osteoporotic and hip fracture events during a median follow-up duration of 10.5 years. This study found that a history of fracture, chronic airway disease, falls, diabetes mellitus and cerebrovascular diseases were significant risk factors for major osteoporotic and hip fractures in older women with osteopenia. The prediction models were developed, using the risk factors, for major osteoporotic and hip fractures, and the performances were AUROCs of 0.73 and 0.75 for major osteoporotic and hip fractures without BMD results.

The representative existing fracture prediction models used in patients with osteopenia are FRAX and Garvan (5). Although both FRAX and Garvan models were good prediction models in patients, they tend to underestimate the risk especially for hip fractures in low-risk patients, as reported in previous studies and was also observed in the Korean version of FRAX (5–8, 14). Furthermore, although secondary osteoporosis was included as a risk factor in FRAX, other critical risk factors such as chronic diseases, cardiovascular and cerebrovascular diseases, type 2 diabetes mellitus, and a history of falls are not included. Even though the risk factors for osteopenia and osteoporosis may be similar (4, 10), but how much they contribute to the risk of fracture in patients with osteopenia might differ. In addition, risk factors might show different effect based on the fracture type. For instance, fall is a key risk factor for hip fracture but not for other fractures (15). Therefore, it is clinically advantageous to demonstrate the risk factors in patients with osteopenia for different fracture types, especially the risk factors such as cerebrovascular diseases or chronic airway disease that are relevant but not included in the existing prediction models.

Cerebrovascular disease is one of the major contributing factors to both major osteoporotic and hip fracture prediction models in this study. The risk of hip fracture in stroke patients was reported to be 4-7 times higher than other major osteoporotic fractures (16). Impairments, such as weakness in the lower extremities, imbalance, loss of autonomic and peripheral sensations, visual impairment, and urinary incontinence after a stroke can significantly contribute to the risk of falls (17). Moreover, the elevation of sclerostin, osteoprotegerin, and FGF23 levels may explain the increased risk of both osteoporotic fractures and cerebrovascular events (18–20). Therefore, this study emphasizes the importance of proactive monitoring and treatment of osteoporosis in post-stroke patients and since cerebrovascular diseases greatly impact the increased fracture risk, managing risk factors of cerebrovascular events is vital in patients with osteopenia.

The history of chronic airway disease (asthma/COPD) was incorporated in the final prediction models. chronic airway disease is associated with low BMD at the spine and hip with an increased risk of vertebral and nonvertebral fractures (21), which might be due to inhalation of corticosteroids, commonly used in patients with asthma/COPD. Corticosteriods are known to decrease bone formation and increase bone resorption, and thus increase the risks of fractures (22, 23). In addition, chronic airway disease itself affect bone health owing to chronic and systemic inflammation (24). Previous studies reported that patients with chronic airflow limitation have significantly elevated inflammatory markers, such as tumor necrosis factor-alpha or c-reactive protein, which have a negative effect on bone (25). Hypercapnia in chronic obstructive lung diseases was associated with increased bone resorption (26). Therefore, this study infers that a history of chronic airway disease could be a valuable risk factor for fracture in patients with osteopenia.

As a major contributing factor, diabetes mellitus, mostly type 2, was included in the final model of both major osteoporotic and hip fractures, with a stronger contribution to hip fractures, which is consistent with other reports (27). This strong contribution might be related to the increased risk of fall caused by autonomic and distal neuropathy, which impairs sensory perception and balance (28). In addition, evidence suggests that impaired insulin metabolism influences bone turnover, leading to decreased bone density and strength (29, 30). Hyperglycemia could lead to increased production of advanced glycosylation end products, which play a vital role in the deterioration of bone quality by inhibiting osteoblastic differentiation (31, 32). Sclerostin levels were substantially increased in patients with diabetes mellitus, associated with inhibition of the Wnt/β-catenin pathway and increased bone fragility (33). Increased cortical porosity, decreased cortical bone strength, obesity, and the effect of antidiabetic medications may be attributed to a higher risk of hip and major osteoporotic fractures (34).

The study did not find obesity or low BMI as a significant risk factor for fractures in women with osteopenia. The association between BMI and fractures is complex and depends on the interaction between BMI and BMD (35). Usually, increased body weight is associated with increased BMD due to the mechanical effect of weight bearing and the metabolic effect of estrogen from adipose tissue (36). However, the effect of obesity on the risk of fractures is controversial (37). In a recent UK Biobank study, an inverted U-shaped association was observed between visceral adipose tissue and risk of fractures in men but not in women (38) which could be attenuated in women due to the differences in the visceral fat distribution and estrogen levels. This partly explains the neutral results observed in this study. On the other hand, low BMI partially correlates with low lean mass, which affects fracture risk (39). However, as this study analyzed only patients with osteopenia, the number of patients with extreme BMI was small. Therefore, BMI might not be a significant factor for fractures in the selected population of older women with osteopenia.

This study has several strengths. This is the largest Asian study on osteopenia confirmed by bone density data. Also, as the data was collected from the nationwide routine health check-up program, it was possible to collect patient information, such as the history of falls, BMI, physical activity, smoking, and drinking, which could not be obtained from the insurance claim database alone. In addition, all participants’ reimbursed healthcare use could be obtained. Therefore, follow-up loss due to transfer or referral to different healthcare providers was unlikely to occur. The models developed in the study were developed based on data with a long-term follow-up in a large nationwide population, which made a 10-year fracture prediction model for osteopenia possible.

The study also had several limitations. Only 66-year-old women were included due to the indication for the national health check-up program. Therefore, the model was created without age and gender information, which may lower the performance. Also, applying the results to high-risk or non-high-risk osteopenia patients, in general, could not be valid since age, sex, and BMD are essential components in the stratification of fracture risks in osteopenia patients. Korean Health Insurance Review and Assessment Service (HIRA) dataset lacks T-score values but only provides in categorical form - normal, osteopenia, or osteoporosis, as the inherent limitation of this dataset, while a more accurate model would be predicted with exact values of bone density. Due to the inherent limitations of the database, we could only present total cholesterol, GTP, and hemoglobin as important laboratory data, while 25-hydroxy vitamin D levels could not be obtained. Although asthma and COPD are different diseases with different etiology, the study analyzed them in combination as chronic airway diseases. Due to the national insurance policy of South Korea, a pulmonary function test (PFT) is necessary to diagnose COPD. However, since PFT may not be readily available in local hospitals, it leads to an excessive diagnosis of asthma even in adult patients because diagnosing asthma does not require PFT according to the policy. Therefore, in a study conducted with the national healthcare database of South Korea, asthma and COPD are often reported as a composite term - asthma/COPD (40–42) because they are hard to distinguish from each other in this unique setting of clinical practice. As the diagnosis of the diseases was operationally defined using diagnostic codes, it could be inaccurate and possibly overestimated. In addition, the information on manufacturer of the DXA machine was not obtain, which could be a major limitation. Since all participants were Korean women, the generalization of the study to other populations should be exercised with caution.

In conclusion, this nationwide cohort study on osteopenia, developed two prediction models of major osteoporotic and hip fractures. The models were developed based on risk factors such as a history of fracture, chronic airway disease, fall, diabetes mellitus, and cerebrovascular disease, with performances of 0.73 and 0.75 in AUROC, respectively. The models have significant clinical importance in the fracture prediction for patients with osteopenia whose fracture burden is rapidly increasing in South Korea and worldwide. However, the prediction models need further validation in external cohorts with various age and gender information.

## Data availability statement

Publicly available datasets were analyzed in this study. This data can be found here: National Health Insurance Services (NHIS) database of South Korea.

## Ethics statement

The studies involving human participants were reviewed and approved by National Evidence-based healthcare Collaborating Agency. Written informed consent for participation was not required for this study in accordance with the national legislation and the institutional requirements.

## Author contributions

SK and AJ equally contributed to this work. SK, All authors read and approved the final version of the manuscript. SK, JY, and JK conceived and designed the study. AJ performed formal analysis. SK wrote the initial draft of the manuscript with assistance from JK. The following drafts were reviewed and edited by SK, AJ, CP, KP, JY, and JK. All authors contributed to the article and approved the submitted version.
