# Supplementary figures and images for: Chronic airway disease as a major risk factor for fractures in osteopenic women: Nationwide cohort study
Source: Front Endocrinol (Lausanne). 2023 Mar 21;14:1085252. doi: 10.3389/fendo.2023.1085252 (PMC10070847; doi:10.3389/fendo.2023.1085252)

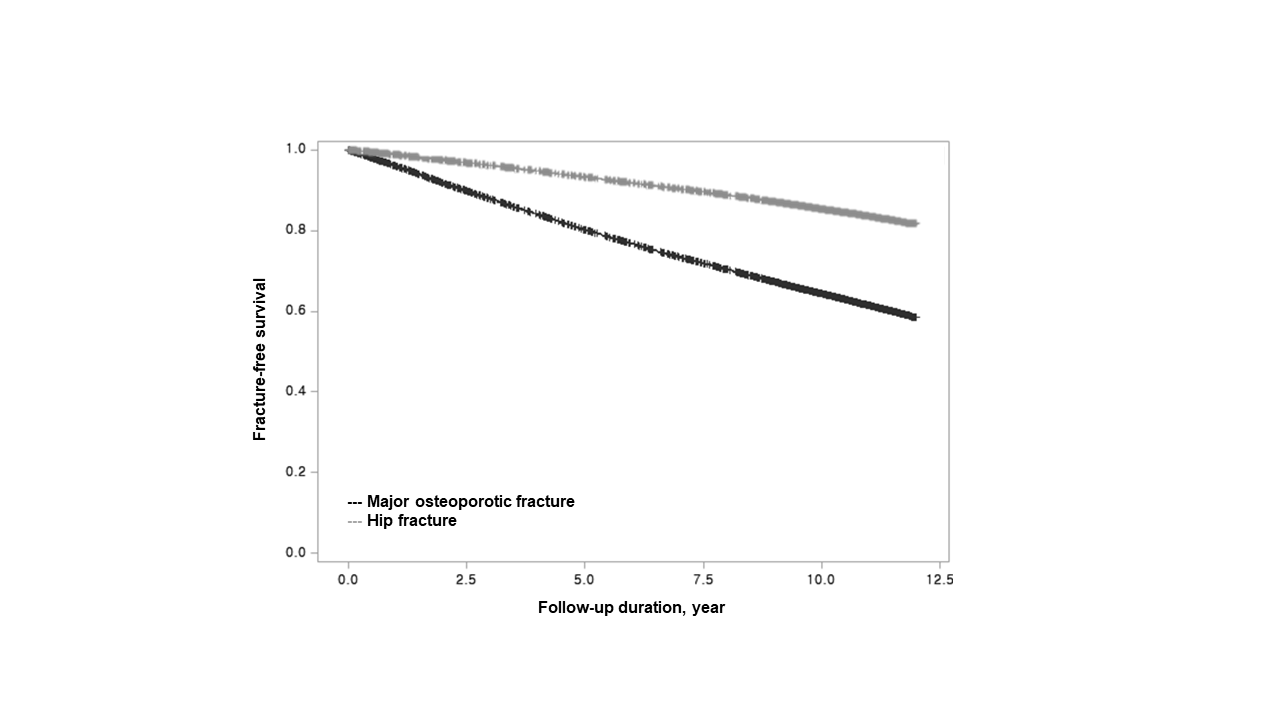

Supplement: Supplementary file 1 [file Image_1.tif]
