# Supplementary material for: Chronic airway disease as a major risk factor for fractures in osteopenic women: Nationwide cohort study
Source: Front Endocrinol (Lausanne). 2023 Mar 21;14:1085252. doi: 10.3389/fendo.2023.1085252 (PMC10070847; doi:10.3389/fendo.2023.1085252)
Supplement: Supplementary file 2 [file Table_1.docx]

**Supplemental Table 1. Model adjustment table for major osteoporotic fracture**

| Variables | Model 1 | | | | | | | | | | | Model 2 | | | | | | | | | | | Model 3 | | | | | | | | |
| --- | --- | --- | --- | --- | --- | --- | --- | --- | --- | --- | --- | --- | --- | --- | --- | --- | --- | --- | --- | --- | --- | --- | --- | --- | --- | --- | --- | --- | --- | --- | --- |
|  | **Beta** | **SE** | | | **HR** | **95% CI** | | | ***p*** | | | **Beta** | | **SE** | | **HR** | | **95% CI** | | ***p*** | | | **Beta** | | **SE** | **HR** | **95% CI** | | | | ***p*** |
| Ever smoker |  | |  |  | | |  |  | |  | | 0.084 | 0.057 | | 1.09 | | 0.97 | | 1.22 | | 0.142 | | 0.083 | 0.057 | | 1.09 | | 0.97 | 1.22 | 0.150 | |
| Current drinker |  | |  |  | | |  |  | |  | | 0.079 | 0.027 | | 1.08 | | 1.03 | | 1.14 | | 0.003 | | 0.082 | 0.027 | | 1.09 | | 1.03 | 1.14 | 0.002 | |
| Low physical activity |  | |  |  | | |  |  | |  | |  |  | |  | |  | |  | |  | | 0.026 | 0.019 | | 1.03 | | 0.99 | 1.06 | 0.167 | |
| History of fall | 0.126 | | 0.026 | 1.14 | | | 1.08 | 1.20 | | <.001 | | 0.128 | 0.027 | | 1.14 | | 1.08 | | 1.20 | | <.001 | | 0.130 | 0.027 | | 1.14 | | 1.08 | 1.20 | <.001 | |
| Diabetes mellitus | 0.120 | | 0.019 | 1.13 | | | 1.09 | 1.17 | | <.001 | | 0.127 | 0.019 | | 1.14 | | 1.09 | | 1.18 | | <.001 | | 0.127 | 0.019 | | 1.14 | | 1.09 | 1.18 | <.001 | |
| Cerebrovascular disease | 0.103 | | 0.035 | 1.11 | | | 1.04 | 1.19 | | 0.003 | | 0.101 | 0.035 | | 1.11 | | 1.03 | | 1.19 | | 0.004 | | 0.101 | 0.035 | | 1.11 | | 1.03 | 1.19 | 0.004 | |
| Chronic airway diseases | 0.177 | | 0.027 | 1.19 | | | 1.13 | 1.26 | | <.001 | | 0.178 | 0.028 | | 1.20 | | 1.13 | | 1.26 | | <.001 | | 0.178 | 0.028 | | 1.20 | | 1.13 | 1.26 | <.001 | |
| History of fracture | 0.847 | | 0.048 | 2.33 | | | 2.13 | 2.56 | | <.001 | | 0.849 | 0.048 | | 2.34 | | 2.13 | | 2.57 | | <.001 | | 0.848 | 0.048 | | 2.34 | | 2.13 | 2.57 | <.001 | |
| Goodness of fi t(Wald) | <.0001 | | | | | | | | | | <.0001 | | | | | | | | | | | <.0001 | | | | | | | | | |
| AUC (C-statistics) | 0.732 (training set), 0.727 (test set) | | | | | | | | | | 0.693 (training set), 0.688 (test set) | | | | | | | | | | | 0.633 (training set), 0.629 (test set) | | | | | | | | | |

SE, standard estimate; HR, hazard ratio; CI, confidence interval; COPD, chronic obstructive pulmonary disease; AUC, area under the curve. Low physical activity was defined as patients who do not have moderate or vigorous physical activity during the past 6 months. Chronic airway diseases include asthma or chronic obstructive pulmonary disease. Multivariate analyses were done. Model 1 adjusted for history of fall, diabetes mellitus, cerebrovascular disease, asthma/COPD, and history of fracture. Model 2 adjusted for smoking and drinking history, addition to model 1. Model 3 adjusted for physical activity, addition to model 2.

**Supplemental Table 2. Model adjustment table for hip fracture**

| Variables | Model 1 | | | | | | | | | | | Model 2 | | | | | | | | | | | Model 3 | | | | | | | | |
| --- | --- | --- | --- | --- | --- | --- | --- | --- | --- | --- | --- | --- | --- | --- | --- | --- | --- | --- | --- | --- | --- | --- | --- | --- | --- | --- | --- | --- | --- | --- | --- |
|  | **Beta** | **SE** | | | **HR** | **95% CI** | | | ***p*** | | | **Beta** | | **SE** | | **HR** | | **95% CI** | | ***p*** | | | **Beta** | | **SE** | **HR** | **95% CI** | | | | ***p*** |
| Ever smoker |  | |  |  | | |  |  | |  | | 0.249 | 0.102 | | 1.28 | | 1.05 | | 1.57 | | 0.015 | | 0.244 | 0.102 | | 1.28 | | 1.05 | 1.56 | 0.016 | |
| Current drinker |  | |  |  | | |  |  | |  | | 0.151 | 0.051 | | 1.16 | | 1.05 | | 1.28 | | 0.003 | | 0.156 | 0.051 | | 1.17 | | 1.06 | 1.29 | 0.002 | |
| Low physical activity |  | |  |  | | |  |  | |  | |  |  | |  | |  | |  | |  | | 0.062 | 0.037 | | 1.06 | | 0.99 | 1.14 | 0.091 | |
| History of fall | 0.175 | | 0.050 | 1.19 | | | 1.08 | 1.31 | | <.001 | | 0.167 | 0.050 | | 1.18 | | 1.07 | | 1.31 | | <.001 | | 0.171 | 0.051 | | 1.19 | | 1.08 | 1.31 | 0.001 | |
| Diabetes mellitus | 0.407 | | 0.035 | 1.50 | | | 1.40 | 1.61 | | <.001 | | 0.416 | 0.036 | | 1.52 | | 1.41 | | 1.63 | | <.001 | | 0.416 | 0.036 | | 1.52 | | 1.41 | 1.63 | <.001 | |
| Cerebrovascular disease | 0.228 | | 0.062 | 1.26 | | | 1.11 | 1.42 | | <.001 | | 0.229 | 0.063 | | 1.26 | | 1.11 | | 1.42 | | <.001 | | 0.230 | 0.063 | | 1.26 | | 1.11 | 1.42 | <.001 | |
| Chronic airway diseases | 0.193 | | 0.053 | 1.21 | | | 1.09 | 1.34 | | <.001 | | 0.198 | 0.053 | | 1.22 | | 1.10 | | 1.35 | | <.001 | | 0.197 | 0.053 | | 1.22 | | 1.10 | 1.35 | <.001 | |
| History of fracture | 0.656 | | 0.086 | 1.93 | | | 1.63 | 2.28 | | <.001 | | 0.653 | 0.087 | | 1.92 | | 1.62 | | 2.28 | | <.001 | | 0.650 | 0.087 | | 1.92 | | 1.62 | 2.27 | <.001 | |
| Goodness of fi t(Wald) | <.0001 | | | | | | | | | | <.0001 | | | | | | | | | | | <.0001 | | | | | | | | | |
| AUC (C statistics) | 0.743 (training set), 0.745 (test set) | | | | | | | | | | 0.708 (training set), 0.708 (test set) | | | | | | | | | | | 0.656 (training set), 0.657 (test set) | | | | | | | | | |

SE, standard estimate; HR, hazard ratio; CI, confidence interval; COPD, chronic obstructive pulmonary disease; AUC, area under the curve. Low physical activity was defined as patients who do not have moderate or vigorous physical activity during the past 6 months. Chronic airway diseases include asthma or chronic obstructive pulmonary disease. Multivariate analyses were done. Model 1 adjusted for history of fall, diabetes mellitus, cerebrovascular disease, asthma/COPD, and history of fracture. Model 2 adjusted for smoking and drinking history, addition to model 1. Model 3 adjusted for physical activity, addition to model 2.
